# Supplementary material for: In Vitro Evaluation of Azoxystrobin, Boscalid, Fentin-Hydroxide, Propiconazole, Pyraclostrobin Fungicides against Alternaria alternata Pathogen Isolated from Carya illinoinensis in South Africa
Source: Microorganisms. 2023 Jun 29;11(7):1691. doi: 10.3390/microorganisms11071691 (PMC10384428; doi:10.3390/microorganisms11071691)
Supplement: Supplementary file 1 [file microorganisms-11-01691-s001.zip › Supplementary Material Figures.pdf]

Supplementary Figure S 1 A - F

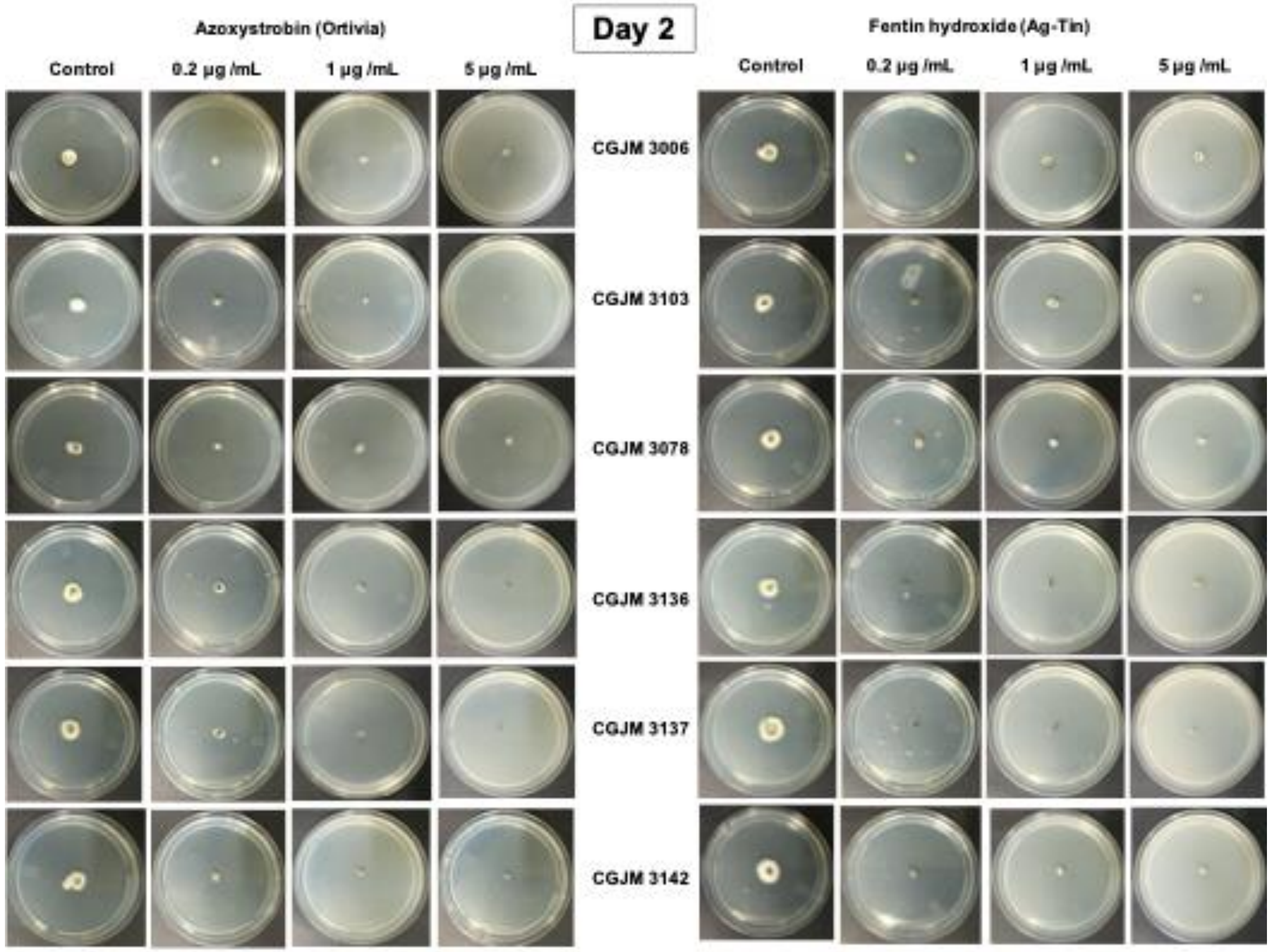

Supplementary Figure S 1A: Effects of azoxystrobin (Ortiva) and fentin hydroxide (AgTin) on mycelial growth of the *A. alternata* isolates. Colony diameter was examined every 2 days post incubation at 25 ± 1 °C for a total of 6 days.

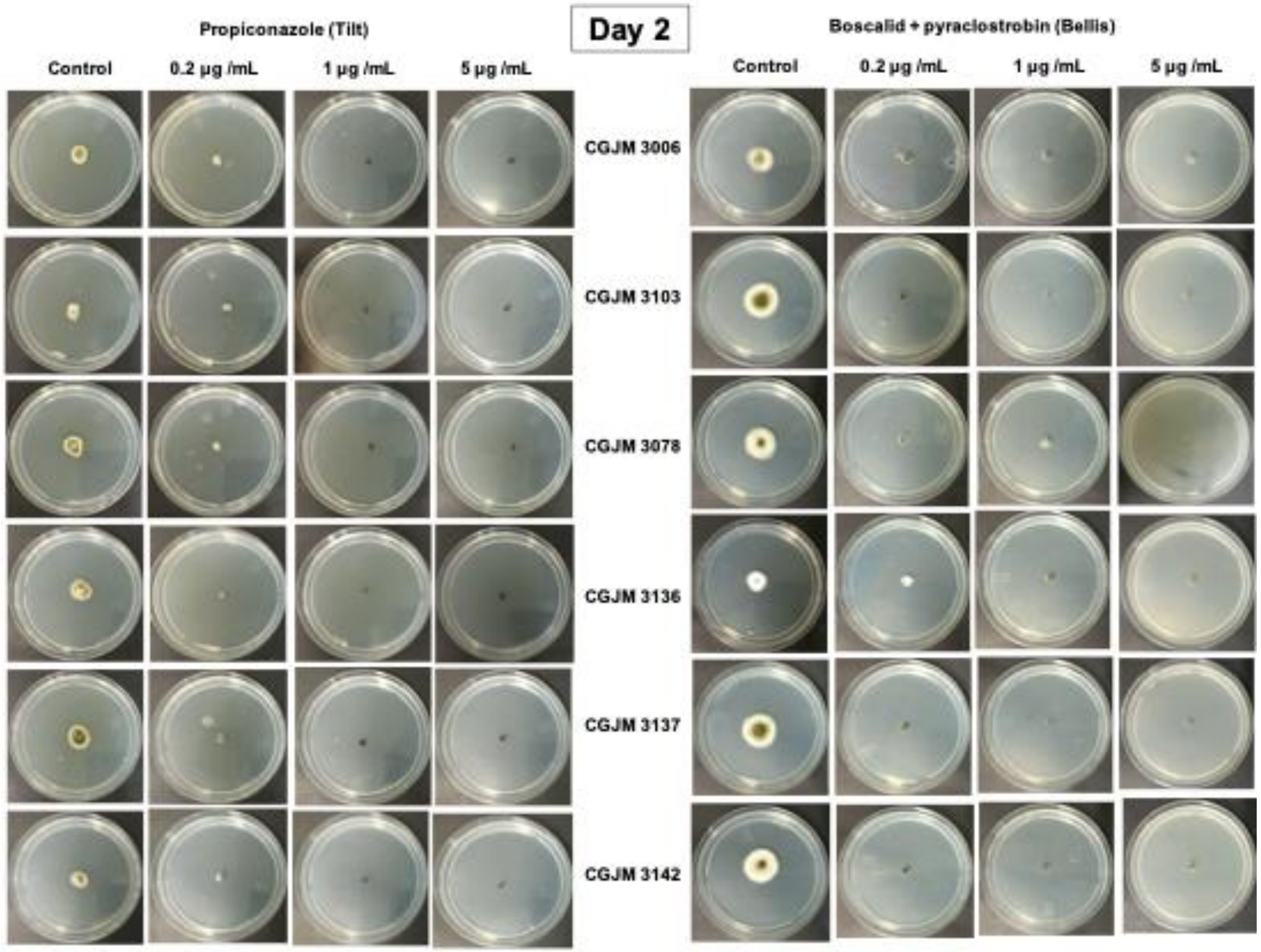

Supplementary Figure S 1B: Effects of propiconazole (Tilt) and boscalid + pyraclostrobin (Bellis) on mycelial growth of the *A. alternata* isolates. Colony diameter was examined every 2 days post incubation at 25 ± 1 °C for a total of 6 days.

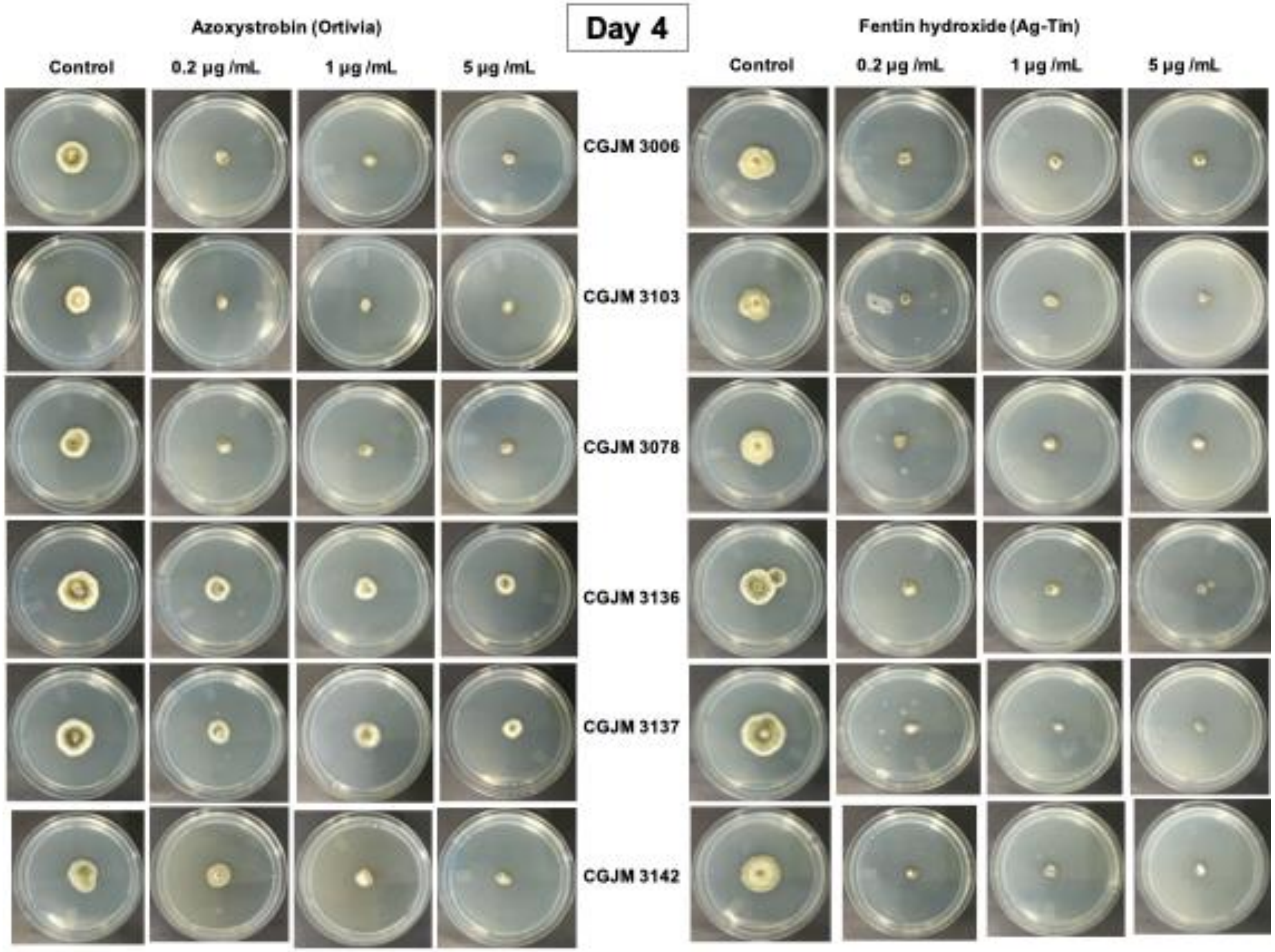

Supplementary Figure S 1C: Effects of azoxystrobin (Ortiva) and fentin hydroxide (AgTin) on mycelial growth of the *A. alternata* isolates. Colony diameter was examined every 2 days post incubation at 25 ± 1 °C for a total of 6 days.

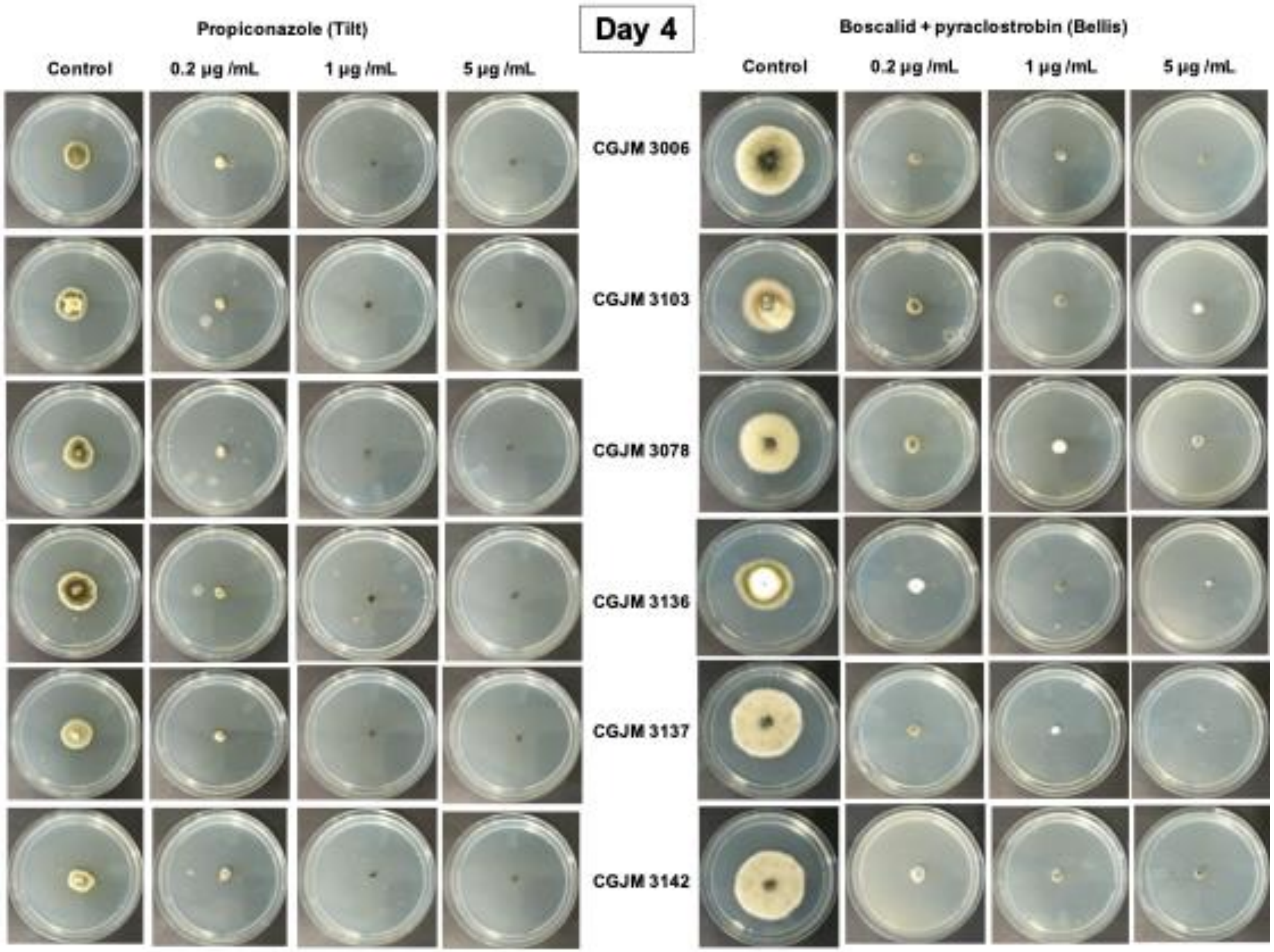

Supplementary Figure S 1D: Effects of propiconazole (Tilt) and boscalid + pyraclostrobin (Bellis) on mycelial growth of the *A. alternata* isolates. Colony diameter was examined every 2 days post incubation at 25 ± 1 °C for a total of 6 days.

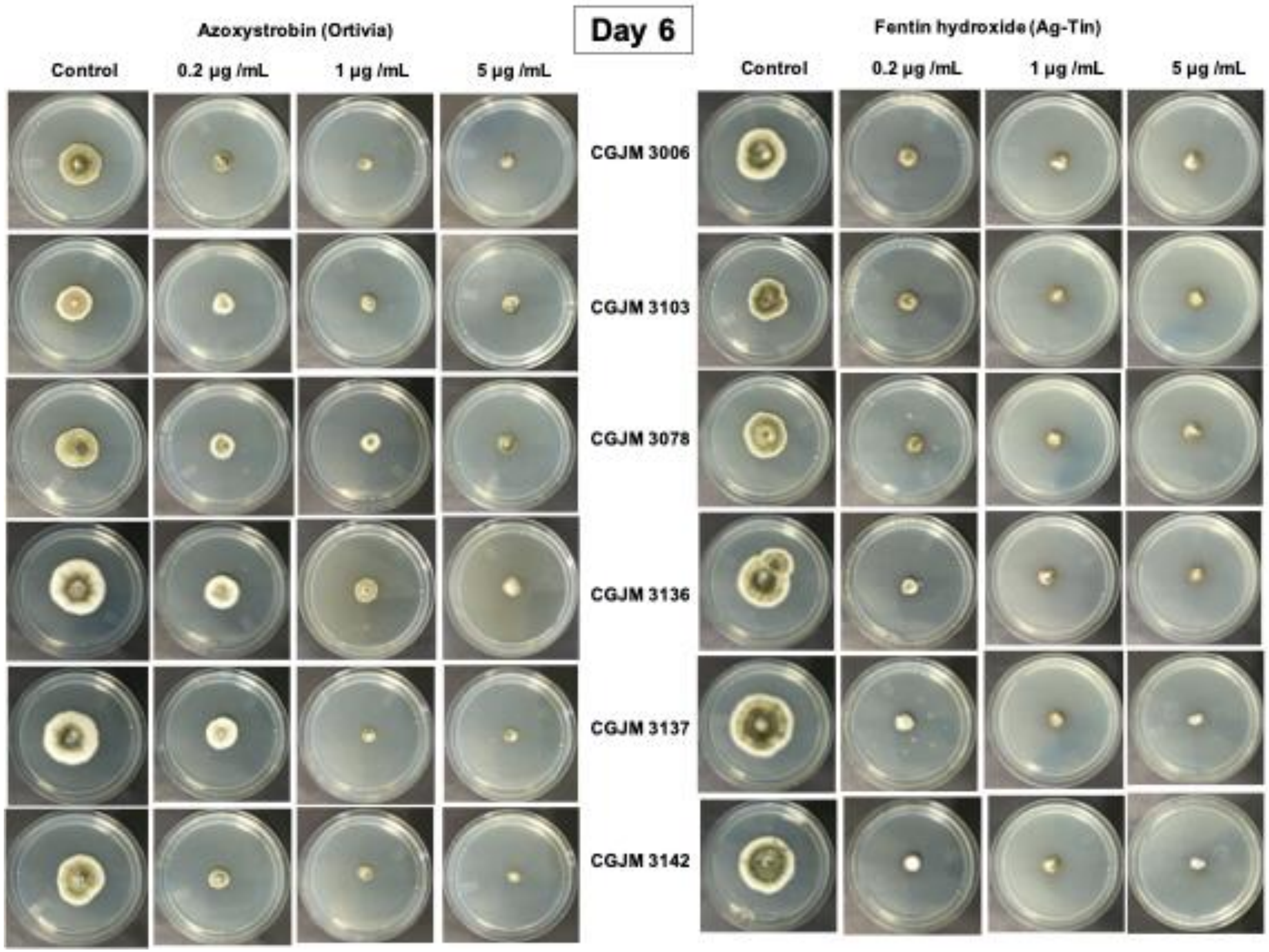

Supplementary Figure S 1E: Effects of azoxystrobin (Ortiva) and fentin hydroxide (AgTin) on mycelial growth of the *A. alternata* isolates. Colony diameter was examined every 2 days post incubation at 25 ± 1 °C for a total of 6 days.

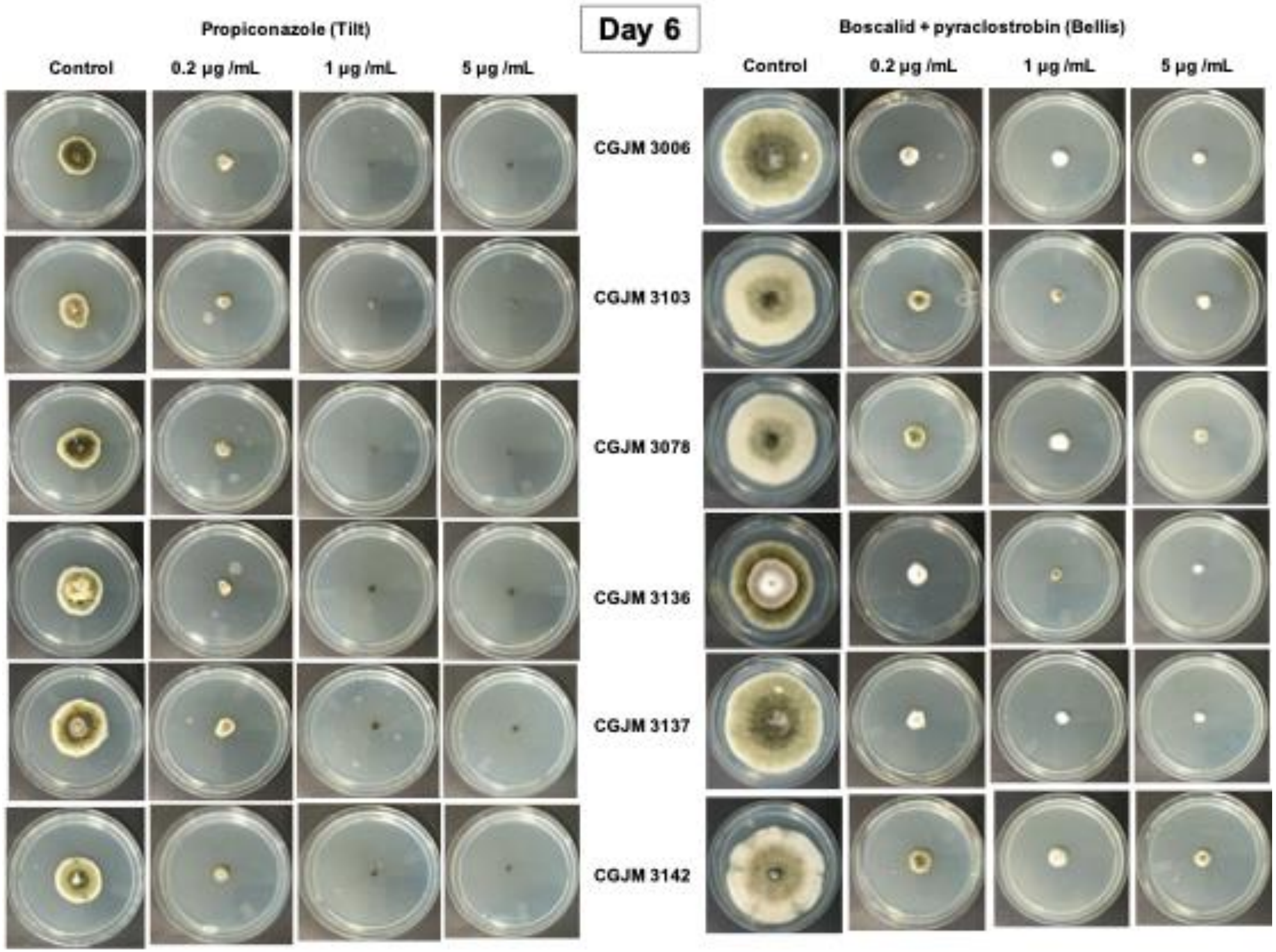

Supplementary Figure S 1F: Effects of propiconazole (Tilt) and boscalid + pyraclostrobin (Bellis) on mycelial growth of the *A. alternata* isolates. Colony diameter was examined every 2 days post incubation at 25 ± 1 °C for a total of 6 days.
