# Supplementary material for: In Vitro Evaluation of Azoxystrobin, Boscalid, Fentin-Hydroxide, Propiconazole, Pyraclostrobin Fungicides against Alternaria alternata Pathogen Isolated from Carya illinoinensis in South Africa
Source: Microorganisms. 2023 Jun 29;11(7):1691. doi: 10.3390/microorganisms11071691 (PMC10384428; doi:10.3390/microorganisms11071691)
Supplement: Supplementary file 1 [file microorganisms-11-01691-s001.zip › Supplementary Material Tables.pdf]

Supplementary Table S1: *In vitro* efficacy detection of six fungicides showing the percentage inhibition of mycelial growth of six *A. alternata* isolates after day 2, 4, and 6 of incubation at 25 ± 1°C.

Inoculation Period	*Alternaria alternata* Isolates	Propiconazole (Tilt)			Azoxystrobin (Ortiva)			Fentin hydroxide (AgTin)			Boscalid + Pyraclostrobin (Bellis)		
		Active Ingredient Concentration (μg mL^{-1})											
		0.2	1	5	0.2	1	5	0.2	1	5	0.2	1	5
		Mean Percentage Inhibition of Colony Growth (%)											
	CGJM3006	48^{C}	100^{a}	100^{a}	52^{z}	65^{o}	71^{j}	40^{H}	44^{F}	58^{t}	33^{J}	37^{I}	46^{D}
	CGJM3078	57^{u}	100^{a}	100^{a}	55^{w}	67^{m}	82^{b}	54^{x}	58^{t}	74^{g}	53^{y}	56^{v}	57^{u}
	CGJM3103	44^{F}	100^{a}	100^{a}	44^{F}	71^{j}	72^{i}	55^{w}	63^{p}	79^{d}	53^{y}	57^{u}	58^{t}
	CGJM3136	70^{k}	100^{a}	100^{a}	37^{I}	65^{o}	81^{c}	59^{s}	62^{q}	70^{k}	49^{B}	56^{v}	62^{q}
Day 2	CGJM3137	68^{l}	100^{a}	100^{a}	37^{I}	67^{m}	78^{e}	58^{t}	65^{o}	73^{h}	53^{y}	57^{u}	59^{s}
	CGJM3142	58^{t}	100^{a}	100^{a}	57^{u}	66^{n}	76^{f}	52^{z}	54^{x}	81^{c}	56^{v}	58^{t}	59^{s}
	α = 0.05	0.001	0.433	0.433	0.001	0.001	0.001	0.001	0.001	0.001	0.001	0.001	0.001
	Control	0.0^{P}	0.0^{P}	0.0^{P}	0.0^{P}	0.0^{P}	0.0^{P}	0.0^{P}	0.0^{P}	0.0^{P}	0.0^{P}	0.0^{P}	0.0^{P}
	Mean	58	100	100	47	67	77	53	58	73	50	54	57
	CGJM3006	56^{G}	100^{a}	100^{a}	60^{D}	66^{w}	75^{n}	61^{B}	64^{y}	80^{i}	53^{I}	57^{F}	64^{y}
	CGJM3078	63^{z}	100^{a}	100^{a}	57^{F}	65^{x}	77^{l}	63^{z}	66^{w}	78^{k}	56^{G}	64^{y}	77^{l}
	CGJM3103	47^{L}	100^{a}	100^{a}	53^{I}	70^{s}	75^{n}	55^{H}	68^{u}	84^{f}	56^{G}	67^{v}	75^{n}
	CGJM3136	71^{r}	100^{a}	100^{a}	38^{N}	68^{u}	84^{f}	66^{w}	73^{p}	84^{f}	53^{I}	63^{z}	75^{n}
Day 4	CGJM3137	70^{s}	100^{a}	100^{a}	40^{M}	65^{x}	82^{h}	40^{M}	65^{x}	82^{h}	56^{G}	62^{A}	73^{p}
	CGJM3142	59^{E}	100^{a}	100^{a}	61^{C}	69^{t}	83^{g}	61^{B}	69^{t}	83^{g}	63^{z}	68^{u}	79^{j}
	α = 0.05	0.001	0.433	0.433	0.001	0.001	0.001	0.001	0.001	0.001	0.001	0.001	0.001
	Control	0.0^{O}	0.0^{O}	0.0^{O}	0.0^{O}	0.0^{O}	0.0^{O}	0.0^{O}	0.0^{O}	0.0^{O}	0.0^{O}	0.0^{O}	0.0^{O}
	Mean	61	100	100	52	67	79	58	67	82	56	64	74
	CGJM3006	58^{c}	100^{a}	100^{a}	66^{cd}	68^{cd}	81^{b}	65^{d}	72^{cd}	89^{a}	58^{e}	74^{b}	78^{ab}
	CGJM3078	68^{bc}	100^{a}	100^{a}	66^{cd}	68^{cd}	80^{b}	66^{d}	71^{cd}	85^{ab}	58^{e}	70^{bc}	79^{ab}
	CGJM3103	67^{bc}	100^{a}	100^{a}	56^{d}	67^{cd}	76^{c}	63^{de}	69^{cd}	85^{ab}	64^{d}	75^{b}	80^{ab}
	CGJM3136	73^{bc}	100^{a}	100^{a}	39^{e}	65^{cd}	86^{a}	70^{cd}	71^{cd}	89^{a}	66^{d}	73^{b}	83^{a}
Day 6	CGJM3137	76^{b}	100^{a}	100^{a}	42^{e}	67^{cd}	84^{ab}	74^{c}	79^{b}	90^{a}	59^{de}	67^{cd}	76^{b}
	CGJM3142	68^{bc}	100^{a}	100^{a}	61^{d}	71^{cd}	84^{ab}	74^{c}	77^{b}	89^{a}	65^{d}	70^{bc}	81^{ab}
	α = 0.05	0.001	0.433	0.433	0.001	0.001	0.001	0.001	0.001	0.001	0.001	0.001	0.001
	Control	0.0^{d}	0.0^{d}	0.0^{d}	0.0^{f}	0.0^{f}	0.0^{f}	0.0^{f}	0.0^{f}	0.0^{f}	0.0^{f}	0.0^{f}	0.0^{f}
	Mean	68	100	100	55	68	82	69	73	88	62	72	80

Control: Mean values for day 2 and 4. Three-way ANOVA differences were considered significant when $p < 0.05$. Different superscript letters indicate significant differences among treatments, according to the Fisher's LSD test

Supplementary Table S2: Three-way ANOVA summary showing the interaction effects of *A. alternata* isolate, fungicide (active ingredient) and concentration of fungicide (µg mL^{-1}) data set of day 2 and 4.

**Inoculation Period**	**Variables**	**D.f.**	**Sum Sq.**	**Mean Sq.**	**F value**	***P*-value**	
Day 2	Isolate	6	77409	12902	135764.3	<0.001	***
	Concentration	2	46508	23254	244701.8	<0.001	***
	Fungicide	5 10	72509	14502	152602.8	<0.001	***
	Isolate x Concentration	30	251	25	264.0	<0.001	***
	Isolate x Fungicide		7706	257	2703.1	<0.001	***
	Concentration x Fungicide	10	15185	1518	15979.1	<0.001	***
	Isolate x Concentration x Fungicide	50	3320	66	698.7	<0.001	***
	Residuals	228	22	0			
Day 4	Isolate	6	95808	15968	296312.4	<0.001	***
	Concentration	2	42315	21158	392614.4	<0.001	***
	Fungicide	5 10	32966	6593	122349.7	<0.001	***
	Isolate x Concentration	30	315	31	584.5	<0.001	***
	Isolate x Fungicide		3773	126	2334.0	<0.001	***
	Concentration x Fungicide	10	7136	714	13242.9	<0.001	***
	Isolate x Concentration x Fungicide	50	3617	72	1342.4	<0.001	***
	Residuals	228	13	0			

Significant codes: 0 '***' 0.001 ($p < 2e-16$) '**' 0.01 *' 0.05 .' 0.1 ' ' 1.

Supplementary Table S3: Concentration (0.2, 1, and 5 μg mL^{-1}) of fungicide sensitivity estimation EC_{50} values that effectively inhibited 50% of mycelial growth for the six *A. alternata* isolates tested.

Isolate	ID	Fungicide	Estimate	Standard error	Lower	Upper
CGJM3006	1	Propiconazole (Tilt)	1.967072	0.006474311	1.952142	1.982002
CGJM3078	2	Propiconazole (Tilt)	1.909757	0.011797829	1.882551	1.936963
CGJM3103	3	Propiconazole (Tilt)	1.912326	0.008772755	1.892096	1.932556
CGJM3136	4	Propiconazole (Tilt)	1.873846	0.015150775	1.838908	1.908783
CGJM3137	5	Propiconazole (Tilt)	1.847434	0.019987422	1.801343	1.893525
CGJM3142	6	Propiconazole (Tilt)	1.910502	0.010092530	1.887229	1.933775
		**Mean**	**1.903490**	0.012045937	1.875712	1.931267

Isolate	ID	Fungicide	Estimate	Standard error	Lower	Upper
CGJM3006	1	Azoxystrobin (Ortiva)	1.491414	0.08241332	1.304982	1.677845
CGJM3078	2	Azoxystrobin (Ortiva)	1.480752	0.07932680	1.301302	1.660202
CGJM3103	3	Azoxystrobin (Ortiva)	1.606251	0.03855435	1.519035	1.693467
CGJM3136	4	Azoxystrobin (Ortiva)	2.698369	0.22738645	2.183985	3.212753
CGJM3137	5	Azoxystrobin (Ortiva)	2.289221	0.10142790	2.059775	2.518667
CGJM3142	6	Azoxystrobin (Ortiva)	1.622359	0.06159138	1.483030	1.761689
		**Mean**	**1.864728**	0.09845003	1.642018	2.087437

Isolate	ID	Fungicide	Estimate	Standard error	Lower	Upper
CGJM3006	1	Fentin hydroxide (AgTin)	1.608458	0.08506523	1.416027	1.800889
CGJM3078	2	Fentin hydroxide (AgTin)	1.536899	0.07788968	1.360701	1.713098
CGJM3103	3	Fentin hydroxide (AgTin)	1.586763	0.08519260	1.394044	1.779482
CGJM3136	4	Fentin hydroxide (AgTin)	1.496285	0.10383956	1.261383	1.731186
CGJM3137	5	Fentin hydroxide (AgTin)	1.480397	0.06684945	1.329173	1.631621
CGJM3142	6	Fentin hydroxide (AgTin)	1.460628	0.07895316	1.282023	1.639232
		**Mean**	**1.528238**	0.08296495	1.340559	1.715918

Isolate	ID	Fungicide	Estimate	Standard error	Lower	Upper
CGJM3006	1	Boscalid + pyraclostrobin (Bellis)	1.648099	0.008497981	1.628875	1.667323
CGJM3078	2	Boscalid + pyraclostrobin (Bellis)	1.626132	0.039253210	1.537335	1.714929
CGJM3103	3	Boscalid + pyraclostrobin (Bellis)	1.552977	0.027521302	1.490720	1.615235
CGJM3136	4	Boscalid + pyraclostrobin (Bellis)	1.527283	0.057273106	1.397722	1.656843
CGJM3137	5	Boscalid + pyraclostrobin (Bellis)	1.556380	0.051413921	1.440073	1.672686
CGJM3142	6	Boscalid + pyraclostrobin (Bellis)	1.505730	0.067244690	1.353611	1.657848
		**Mean**	**1.569434**	0.04186737	1.474723	1.6641446
